# ABCA7 Loss of Function Variants Disrupt Neuronal Lipid Metabolism and Respiration

**DOI:** 10.1002/alz70855_103044

**Published:** 2025-12-23

**Authors:** Djuna K Von Maydell, Shannon Wright, Colin Staab, Ping‐Chieh Pao, Oisin King, Julia Maeve Bonner, Andrea Spitaleri, Liwang Liu, Ching‐Chi Chiu, Daniel Leible, Chung Jong Yu, Aine Ni Scannail, Mingpei Li, Carles A Boix, Hansruedi Mathys, Guillaume Leclerc, Gloria Suella Menchaca, Gwyneth Welch, Agnese Graziosi, Noelle Leary, George Samaan, Manolis Kellis, Li‐Huei Tsai

**Affiliations:** ^1^ Massachusetts Institute of Technology, Cambridge, MA, USA; ^2^ University of Milan, Milan, 20122, Italy; ^3^ University of Pittsburgh, Pittsburgh, PA, USA

## Abstract

**Background:**

Rare loss‐of‐function variants in the lipid transporter ABCA7 are among the strongest genetic risk factors for Alzheimer's disease. However, the mechanisms by which these variants increase risk and their relevance to the larger at‐risk population remain largely unknown, limiting therapeutic development.

**Method:**

To investigate this, we performed single‐nuclear RNA sequencing on brain samples from a rare cohort of ABCA7 loss‐of‐function variant carriers (12 carriers and 24 matched non‐carrier controls). We compared these transcriptional results with available *post‐mortem* data from ABCA7 p.Ala1527Gly carriers (135 carriers and 240 controls). This analysis was further complemented by molecular dynamic simulations and functional studies using iPSC‐derived neurons and neurospheroids.

**Results:**

In the human brain, excitatory neurons, which express the highest levels of ABCA7, displayed transcriptional disruptions in lipid metabolism, mitochondrial function, and synaptic signaling. Overlapping transcriptional changes were identified in carriers of the common AD‐risk variant ABCA7 p.Ala1527Gly, predicted by molecular dynamic simulations to disrupt ABCA7 structure.

We confirmed similar transcriptional changes in iPSC‐derived human neurons carrying ABCA7 loss‐of‐function variants. These cells showed triglyceride buildup, disrupted phosphatidylcholine metabolism, and impaired mitochondrial function, especially in regulating membrane potential and supporting respiration. Treatment with CDP‐choline, a dietary precursor for phosphatidylcholine synthesis, restored mitochondrial function, reversed many transcriptional defects, and reduced amyloid‐β pathology in ABCA7 loss‐of‐function neurons.

**Conclusion:**

These findings suggest that phosphatidylcholine disruption may underlie metabolic and pathological defects in the context of impaired ABCA7, with potential relevance to a broader population. In line with a growing body of evidence, these findings implicate lipid dysfunction in Alzheimer's disease etiology and suggest therapeutic avenues for a subset of at‐risk individuals.

